# A Brief Survey of Recent Advances and Methodologies for the Security Control of Complex Cyber–Physical Networks

**DOI:** 10.3390/s23084013

**Published:** 2023-04-15

**Authors:** Ying Wan, Jinde Cao

**Affiliations:** 1 Department of Systems Science, School of Mathematics, Southeast University, Nanjing 210096, China; 2 Yonsei Frontier Lab, Yonsei University, Seoul 03722, Republic of Korea

**Keywords:** complex cyber–physical networks, secure control, hybrid attack, denial-of-service (DoS) attack, false data injection (FDI) attack, proactive defense

## Abstract

Complex cyber–physical networks combine the prominent features of complex networks and cyber–physical systems (CPSs), and the interconnections between the cyber layer and physical layer usually pose significant impacts on its normal operation. Many vital infrastructures, such as electrical power grids, can be effectively modeled as complex cyber–physical networks. Given the growing importance of complex cyber–physical networks, the issue of their cybersecurity has become a significant concern in both industry and academic fields. This survey is focused on some recent developments and methodologies for secure control of complex cyber–physical networks. Besides the single type of cyberattack, hybrid cyberattacks are also surveyed. The examination encompasses both cyber-only hybrid attacks and coordinated cyber–physical attacks that leverage the strengths of both physical and cyber attacks. Then, special focus will be paid to proactive secure control. Reviewing existing defense strategies from topology and control perspectives aims to proactively enhance security. The topological design allows the defender to resist potential attacks in advance, while the reconstruction process can aid in reasonable and practical recovery from unavoidable attacks. In addition, the defense can adopt active switching-based control and moving target defense strategies to reduce stealthiness, increase the cost of attacks, and limit the attack impacts. Finally, conclusions are drawn and some potential research topics are suggested.

## 1. Introduction

**Figure 1:**
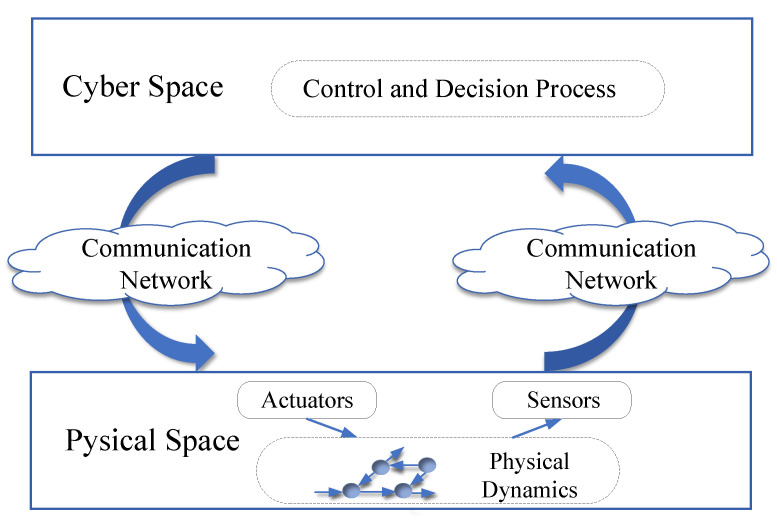
General framework of complex cyber–physical networks.

The integration of complex network principles with those of cyber–physical systems (CPSs) results in the formation of complex cyber–physical networks, in which the interactions between the cyber and physical components exert a substantial influence on the network’s regular functioning [1]. The field of current research on complex cyber–physical networks has evolved to encompass a holistic perspective that considers both the physical infrastructure and cyberspace components. Figure 1 depicts a general framework for such complex cyber–physical networks, where cyber and physical spaces are tightly interconnected. As the information age progresses, an increasing number of vital infrastructures can be considered complex cyber–physical networks due to such integration of cyber elements with the physical layer, such as power grid systems, transportation networks, the Internet of things (IoT), etc. The social and economic significance of these complex cyber–physical networks has become increasingly pronounced, leading to an enormous increase in research from a variety of fields [2,3,4].

The issue of high reliability is a paramount consideration and a significant challenge in configuring complex cyber–physical networks. The successful deployment of these networks into social applications, such as smart city construction and medical care systems, dramatically depends on improvements in reliability and safety. However, integrating cyber-communication components also presents a risk of malicious attacks via cyber channels. Some most notable cyberattacks on CPSs in recent decades include but are not limited to the following issues. Since its discovery in 2010, Stuxnet has gained recognition for its disruptive impact on Iran’s nuclear program. The malware was designed to specifically target the Supervisory Control and Data Acquisition (SCADA) systems, resulting in substantial harm to the program [5]. The Ukraine power outage in 2015 was the first known successful cyberattack on a power grid, causing widespread power outages in Ukraine. The attack was carried out by malware that infiltrated the control systems of power distribution companies, causing widespread blackouts [6]. By continually propagating to poorly configured Internet of Things (IoT) devices, Mirai produces a distributed denial of service (DDoS) to a group of target servers. Notably, one in October 2016 targeting service provider Dyn knocked hundreds of websites down for several hours, including Twitter, Netflix, Reddit, and GitHub [7]. The SolarWinds hack was a major cyberattack that occurred in 2020 [8], where hackers deployed malicious code into the company’s Orion software, which was widely used by various organizations globally. The attack caused widespread disruption and raised significant concerns about the security of supply chain software and the potential for future attacks. These incidents emphasize the importance of enhanced security safeguards to protect these systems from cyber threats and remind people to have in-depth analysis and take proactive defense methodologies.

Current computer and information technology methods focus the security issue mainly on cyber-protection methods such as encryption, building firewalls, or developing antivirus software [9]. These methods are primarily based on the fundamental confidentiality, integrity, and availability (CIA) triad rules [10]. Although these works can defend against certain specific network communication attacks, they are restricted mainly to the information space and only consider data security and network communication security, with the primary goal of protecting data privacy and normal network communication. Furthermore, how the considered CPSs can survive to function in a relatively satisfactory way is rarely emphasized therein. Deep integration with physical space and comprehensive considerations on the complex dynamics of physical systems still need to be included. The implementation of cybersecurity measures derived from information and communication technology would result in a reasonable solution that may appear plausible. However, the unique characteristics of complex cyber–physical networks mean that it is insufficient to consider the CIA view of the system solely. The safeguard of complex cyber–physical networks encompasses not only the protection of data and communication, but also the assurance of secure and unencumbered functioning of the physical systems with interconnected agents.

Table 1 illustrates some recent surveys or overviews on the security issues in multi-agent systems (MASs) [11,12,13], networked control systems [14,15], or CPSs [16]. The authors note that, at the time of writing, there is a scarcity of comprehensive surveys that examine the state-of-the-art advances and novel methodologies in secure control for complex cyber–physical networks. Such surveys should consider both realistic and advanced attacks, as well as proactive defense mechanisms that provide both pre-emptive protection and post-recovery or mitigation strategies.

**Figure 2:**
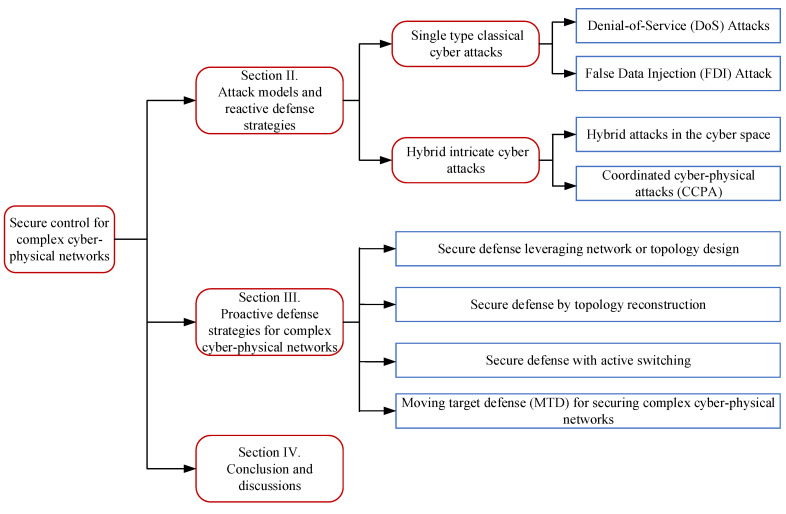
The main structure of this survey.

In light of the motivations discussed above, this paper is concerned with the most recent advances in single-type classical cyberattacks, which mainly focus on DoS attacks, FDI attack, and the newly emerged hybrid cyberattacks that can be more intricate, collusive, and intelligent. Hybrid attacks that only happen in cyberspace and the coordinated cyber–physical attack that combines the advantages of the physical attack and cyberattack are delved into. Existing reactive defense strategies are first given, where the defense strategies are mainly based on the specific characteristics of the cyberattacks, usually with control parameters directly related to the attack indexes. This indicates that the defense is carried out reactively. Although reactive defense methods, such as attack intrusion detection, have made substantial efforts to safeguard a system or network, their reactive nature is limited when confronted with more persistent, sophisticated, and unsuspected attacks. Thus, subsequently, some recent secure control methodologies of a proactive nature are surveyed based on topology and control views. The topology design allows the defender to resist potential attacks beforehand, while the reconstruction method can contribute to a rapid recovery from unavoidable attacks. Active switching-based control and moving target defense ideas are also reviewed. The defender can proactively choose alternative control policies such that the stealthiness is reduced, the attack budget is increased, and the attack impact is mitigated. The organization of the survey is illustrated in Figure 2.

## 2. Attack Models and Reactive Defense Strategies in Complex Cyber–Physical Networks

### 2.1. Single Type Classical Cyberattacks

#### 2.1.1. Denial-of-Service (DoS) Attacks

Attackers with inadequate knowledge of the target system may resort to DoS attacks that prevent sending control and measurement data packets. DoS attacks aim to impede the normal operation of a system by inundating its communication channel with excessive amounts of data traffic or requests, thus leading to the disruption of normal functioning and resulting in significant consequences if not addressed in a timely manner. To address these issues, the first step one needs to take is to propose proper ways to define attack occurrences.

In [18], the authors characterize the occurrence of DoS signals using the Bernoulli process and time-homogeneous Markov chain. A time-inhomogeneous Markov chain was also introduced, so the network difference between consecutive packet transmission instants can be incorporated. Furthermore, noting that, in contrast to traditional packet losses that are described as random occurrences, a stochastic characterization of DoS attacks would be intrinsically restrictive since it would fail to reflect an attacker’s hostile and sophisticated feature. In [19], the authors suggested a model that permits DoS attacks to occur in any sequence provided that the total duration of attacks in a certain time interval be limited by some function of the interval’s length. The following assumption is introduced for describing DoS attacks regarding the total attack interval:

**Assumption** **1.** *There exist nonnegative constant κ and positive constant τ such that DoS sequence $\left\{h_{n}\right\}$, n∈N satisfying:*

(1) $$\left|\Xi \left(t\right)\right|\leq \kappa +\frac{t}{\tau },\;\;\forall t\geq 0,$$

*where Ξ(t) denotes the total interval of DoS attacks from t=0 up to the current time t.*

Afterwards, it was found that, in order to resist and tolerate DoS attacks to maintain stability, suitable conditions must be imposed on both DoS frequency and duration [20]. By utilizing the concept of average dwell-time in hybrid systems [21], the following assumptions on DoS signals were proposed:

**Assumption** **2** (DoS Frequency)**.** *There exist nonnegative constant η and positive constant $\tau_{D}$ (greater than the minimum possible sampling rate) such that*

(2) $$n\left(\tau ,t\right)\leq \eta +\frac{t-\tau }{\tau_{D}},$$

*for all τ,t≥0 with any τ satisfying: τ≤t, where n(τ,t) denotes the number of DoS off/on transitions in time interval [τ,t].*

**Assumption** **3** (DoS Duration)**.** *There exist nonnegative constant κ and positive constant T such that*

(3) $$\left|\Xi \left(\tau ,t\right)\right|\leq \kappa +\frac{t-\tau }{T},$$

*for all τ,t≥0 with any τ satisfying τ≤t, where Ξ(τ,t) denotes the set of time instants with denied communication within [τ,t]. Note that (2) is the generalized form of (1) and can effectively deal with a system whose dynamics contain disturbances [22].*

Some interesting resilient control results on DoS attacks have been reported in recent years. It was shown in [23] that consensus in MASs could be attained with randomized transmissions if the average attack time is constrained, irrespective of attack frequency. In [24], the authors studied the consensus tracking problem under DoS attacks that occurred in the communication network connecting the physical and cyber layers, and those launched among cyber communication links within the cyber layer were both considered. The timely updating of control inputs is impacted by the first type of attack, while the second type alters the link weights in the cyber communication graph. The fully distributed cooperative control of MASs in the presence of distributed denial-of-service (DDoS) attacks was further explored in [25]. In [26], the secure consensus problem for multiple-input-multiple-output (MIMO) linear MASs subject to DoS attacks was investigated. An unknown input observer (UIO) was newly introduced, based upon which a resilient consensus controller was designed with only relative outputs.

Another method to model DoS attacks was given in [27], where power-constrained pulse-width modulated (PWM) DoS signals were introduced in single-input controllable linear systems, and the attacker repeats cycles of jamming and sleeping as the following rules:

(4) $$u_{\mathrm{jmd}}\left(t\right)=\left\{\begin{array}{c} 0,\;T^{n-1}\leq t\leq T^{n-1}+T_{\mathrm{off}}^{n-1},\\ 1,\;T^{n-1}+T_{\mathrm{off}}^{n-1}<t<T^{n}, \end{array}\right.$$

Concerning the dynamic interactions between the attacker and defender, researchers have obtained optimal DoS attack and defense schemes in various problem settings with a game-theoretic approach. For instance, the interactive process of choosing to communicate and attacking time was investigated for both the attacker and the sensor [28] in the context of energy restrictions:

(5) $$\sum_{k=1}^{T}\gamma_{k}\leq M,\;\;\sum_{k=1}^{T}\lambda_{k}\leq N,$$

where $\gamma_{k}=1$ indicates that the system sends the data packet at time *k*, $\lambda_{k}=1$ denotes that the attacker launches a DoS signal at time *k*, and *M* and *N* give the upper bounds of the energy limitations of the defender and the attacker, respectively. With the use of a game-theoretic framework, it was demonstrated that the best action to take for each side results in a Nash equilibrium in a zero-sum game. A Stackelberg game with SINR-based formulation for wireless networked control systems was also considered by [29]. Strong and normal types of DoS jamming signals were studied in [30], where attacks can cut off communication edges in the network and divide the agents into clusters. Meanwhile, the defender seeks to recover selected edges with increased transmission power. The repeated games between the attacker and the defense were derived, where the two players’ best strategies were determined by a rolling horizon and based on the cluster sizes.

By the above brief check of the related works on DoS attacks from a control perspective, one can know that inadequately informed attackers may opt to carry out such DoS attacks, which hinder the transmission of control and measurement data packets. These attacks are designed to hinder the standard operations of a system by overwhelming its communication channel with excessive data traffic or requests. Consequently, this disruption of regular functioning may have significant consequences if left unattended. Further research on this topic may focus on developing advanced techniques for accurately modeling intelligent DoS attacks, thus detecting and mitigating their effects. These methods can include the integration of machine learning algorithms to identify and mitigate attack instances in real time. Developing strategies to enhance system resilience to such attacks needs further exploration, which may include deploying redundant systems, backup communication channels, or communication-saving secure control strategies to tackle potential DoS attacks.

#### 2.1.2. False Data Injection (FDI) Attack

A false data injection (FDI) attack is the type of cyberattack that injects false data into the vital measurements in the system intending to disrupt or alter the normal operation of the original system [31,32,33]. Note that replay attacks are also classified as special deception attacks since they use recorded data to falsify current transmission states [34]. It should be noticed that the impacts of an FDI attack can be amplified in complex cyber–physical networks. Thus, much effort should be devoted to investigating FDI attacks therein. Many interesting works have been conducted in recent years, including attack detection [35,36,37,38,39], resilient control strategies [40,41,42,43], and survey articles [15,44,45]. The following are some representative works on this subject since this subsection is not the primary focus; for more details, one may refer to the specialized survey paper on this topic listed above.

In [36], the authors considered the FDI attack in the DC microgrids with the following form:

(6) $$y_{i,j}^{c}\left(t\right)=y_{j}\left(t\right)+g\left(t-T_{a}\right)\phi_{i,j}\left(t\right),$$

where $\phi_{i,j}\left(t\right)$ is the injected vector designed by the attacker and $g(t-T_{a})$ is the step function with delay $T_{a}$, which means that the attack starts at time instants $T_{a}$ and remains silent before $T_{a}$. The full-order UIO-based detectors were utilized for detection and, based on the estimated average point of common coupling voltage (PCC) voltage, a distributed countermeasure against cyberattacks was also given. In [37], the authors proposed a FDI detection strategy based on secure federated deep learning, based on which, a collaborative training of a detection model is performed using data from all nodes while maintaining the privacy of the data by retaining it locally throughout the training process. In [38], a novel method for detecting FDI attacks in a distributed manner was proposed, requiring only communication between neighboring nodes. The feedback mechanism, which utilizes a dynamic time-wrapping distance, was designed to handle delayed communication and varying attack signals. In [39], the authors designed a FDI detection approach relying on a graph neural network that considers changes in a power grid architecture. Using a gated graph neural network (GGNN), the approach collects spatial information from both the power grid structure and operational data. An attention technique was used to allocate aggregation weights to neighboring nodes to improve node representation.

Recently, in the resilient control against FDI attacks, an attack-free protocol was designed in [40] for cooperative tracking with only relative output measurement. FDI attack can be well resisted in such a setting since the proposed protocol requires no communication information exchange among neighboring agents. In [41], the authors studied the resilient control problem for a DC microgrid in current sharing and voltage restoration. The FDI attack was modeled with impulsive signals at specific time instants that aim to manipulate the set points. Using the stability analysis from the hybrid system, the sufficient condition was derived by using the average dwell time of FDI attacks therein. The resilient consensus problem in DC microgrids under FDI attacks was tackled in [42], where FDI attacks can be isolated by the employed distributed bank of sliding mode observers and the impacts of attack in the control protocol can be eliminated.

Through the above brief review on FDI attacks, one can see that this type of attack differs from DoS attacks in both goals and methods of operation. An FDI attack usually alters or disrupts the regular operation of a system by injecting false data, and the attacker’s goal is to cause harm to the system or cause it to behave in unexpected ways. In contrast, a DoS attack aims to disrupt the normal functioning of a system by overwhelming the communication channel with data traffic or requests. Both FDI and DoS attacks may have severe consequences for the operation of control systems, system misbehavior, physical equipment damage, and financial losses. Compared with DoS attacks, FDI attacks can be designed to be more sophisticated and highly stealthy. Some existing works pose certain restrictions on the mathematical forms, attack indexes, or attack abilities for FDI attacks. However, these assumptions may face certain limitations when confronted with more powerful or intelligent attacks. Thus, more effort is still needed to immunize complex cyber–physical networks under such attacks from new perspectives.

### 2.2. Hybrid Intricate Cyberattacks

#### 2.2.1. Hybrid Attacks in the Cyberspace

Hybrid attacks with various types of cyberattacks may realize more attack goals simultaneously and have more extensive attack impacts when compared with the signal type of attacks. A batch of results appeared concerning the hybrid attacks that combine the DoS and FDI attacks.

In [43], the authors considered the distributed event-based control strategy for thermostatically controlled loads (TCLs) to realize fair sharing of both power states and comfort states. The communication network of TCLs is subjected to hybrid attacks composed of DoS and FDI attacks, whose attacking strategy is blocking the information transmission even though the event-triggered condition is satisfied and tampering with the values of control inputs, respectively. Sufficient conditions regarding the attack parameters, including the DoS duration and frequency indexes, and the FDI degree parameters were derived to achieve a fair sharing goal. Furthermore, the collusive effects of such hybrid cyberattacks and the overall effects were revealed. Mean-square exponential consensus under event-triggered impulsive control strategy was studied in [46] for MASs with nonlinear dynamics and under hybrid attacks, where Bernoulli distribution variables were introduced to describe the successful DoS attack and a successful FDI attack. Sufficient conditions were given to ensure the consensus of the MAS and a lower bound of the impulsive sequence was provided to avoid the Zeno behaviors. The secure control of the connected vehicle system was investigated in [47], with DoS attacks described by attack frequency and length rate and FDI attacks with unknown potential deception attack signals and occurrence probabilities. The conditions of controller gains and the upper bounds of DoS attack indexes were calculated to ensure the asymptotic tracking of follower vehicles. The synthesis of hybrid cyberattacks was studied in [48] for the network control system with heterogeneous agents, and the attack is a combination of FDI and DoS attacks. The attack synthesis problem for choosing proper injection attack vector $u^{a}\left(t\right)$ and vehicle selection matrix $B^{a}$ was given as

(7) $$\begin{array}{cc} \mathrm{find}\;\; & B^{a},u^{a}\left(t\right),\\ \mathrm{such}\;\mathrm{that}\;\; & \dot{x}\left(t\right)=A_{G(t)}x\left(t\right)+B^{a}u^{a}\left(t\right),\\ \; & \exists t^{*}\geq t_{1}\;\;\mathrm{where}\;\;{∥x_{i}\left(t^{*}\right)-x_{j}\left(t^{*}\right)∥}_{2}\geq d^{*},\\ \; & ∥u^{a}{\left(t\right)∥}_{2}\leq \rho \;\;\mathrm{for}\;\mathrm{all}\;\;t\geq t_{1}, \end{array}$$

where $A_{G(t)}=\mathrm{diag}{\left\{A_{i}+B_{i}K_{ii}\right\}}_{i\in V}+B_{i}K_{ij}⊗L_{G(t)}$, ρ denotes the FDI budget and $d^{*}$ denotes the targeted separation distance of the attacker. To disrupt the formation, a sustained DoS attack was also carried out on certain agents such that underlying communication links will be disrupted into a disconnected graph. It was revealed in the example of unmanned aerial vehicles (UAVs) that a combined attack could lead to more severe performance degeneration, and both the formation and trajectory tracking goals failed. Recently, several researchers also explored the hybrid attacks composed of DoS and deception attacks for load frequency control (LFC) issues in smart grids [49,50].

Some published works further consider hybrid attacks, where at least three types of cyberattacks were comprehensively considered, for instance, a hybrid attack model composed of stochastic deception attacks, replay attacks, and DoS attacks all occurring in the communication network [51].

Sometimes, hybrid attacks with attacks in more than one channel may happen in CPSs. For example, the sensor-to-controller and controller-to-actuator channels are both subjected to cyberattacks. Differing from traditional observer-based control problems for complex dynamical networks and multi-agent systems, the scenario was explored that the communication channels for controllers and observers were both subjected to malicious attacks that aim to destroy the communication links among the agents [52]. The attacks on different communication networks are assumed to be independent and effective security control strategies with properly selected feedback gains, and coupling strengths were derived. Later on, the case where DoS attacks can occur in the controllers’ and observers’ communication networks in different time instants, namely asynchronous attacks, was further explored in [53]. Furthermore, regarding asynchronous attacks, [54] studied the asynchronous attacks made of connection attacks on network connections and DoS attacks in control channels for complex networks. In [55], the authors studied the consensus for two-layered MASs subject to asynchronous attacks on communication edges, where the communication edges among the leaders and those among the follower layer may be attacked at different moments. Two switching signals were introduced to characterize such asynchronous attacks. When combined with a new type of multiple Lyapunov function (MLF) based on a bisection search method, criteria were established under which the node-to-node consensus error will asymptotically converge into a bounded set. In [56], the authors considered the hybrid attack composed of deception attacks and DoS attacks, which occur in the sensor-to-observer channel and observer-to-controller channel, respectively. Dynamic event-triggered schemes were given, and sufficient conditions were derived to guarantee the resulted closed-loop system can be asymptotically mean-square stable with a prescribed $H_{\infty }$ performance. The hybrid attack composed of distributed DoS (DDoS) and FDI attacks was considered in [57] for discrete-time CPSs, where DDoS attack causes delays with Bernoulli distributions in both sensor-to-controller channel and controller-to-actuator channel. Due to its straightforward construction, low cost, and high-impact features, the DDoS attack may be more easily organized by utilizing many hacked devices. An event-triggering control scheme was designed for the compromised CPSs with random delays in measurements and actuation signals.

From the attacker’s view, the optimal scheduling for hybrid attacks that are composed of DoS attacks and stealthy attacks on remote state estimation was studied in [58], with the attacker aiming to maximize average error with stated energy restriction. The ideal hybrid attack schedule is derived theoretically after examining average error under carefully determined cases.

#### 2.2.2. Coordinated Cyber–Physical Attacks

The previous examination centered on hybrid attacks that occur exclusively in cyberspace. It is essential to acknowledge, however, the emergence of a novel form of attack referred to as coordinated cyber–physical attacks (CCPAs), in domains such as smart grids, has become increasingly prevalent in recent years, characterized by the integration of both cyber and physical attacks. These attacks are distinctive due to their ability to mask physical attacks with cyber assaults, leading to severe and often undetectable consequences. The 2015 attack on the Ukrainian electrical grid is a notable example of the destructive effects of CCPAs. The physical attack involved opening circuit breakers and caused widespread power outages affecting 225,000 consumers. Telephonic flooding and server wiping further exacerbated the crisis by concealing the extent of the attack and prolonging the outages [59,60,61].

CCPA can be classified into two categories based on the nature of physical attacks. The first category encompasses stealthy physical attacks that are initiated with the goal of compromising components of power systems, followed by a cyberattack aimed at disguising the physical attack, resulting in a covert CCPA. The second category encompasses cyber–physical attacks that may not be stealthy and the attackers aim to achieve a combined effect that is more pronounced than the sum of their individual actions, regardless of the detectability of the physical attack. It is important to highlight that both of these categories of CCPA can result in more severe consequences than single attacks. This hybrid attack mode has garnered the attention of researchers, leading to the development of various countermeasures.

A comprehensive framework for modeling cyber–physical attacks in dynamic power networks was presented in [60]. Research has shown that even local cyberattacks, executed with limited network information, can effectively mask transmission line failures [62,63]. In [61], the authors conducted a study on two specific types of cyber–physical attacks, replay and optimized attacks, in smart grid systems. The authors demonstrated that these attacks can effectively conceal transmission line failures by replaying meter readings and manipulating PMU measurements. Additionally, the necessary capability of an attacker to carry out these attacks was established and, based on this, countermeasures to detect such attacks were proposed.

On the one hand, in the works [61,62,63], the physical attack was conducted first and then cyberattacks were performed subsequently to cover the single line outage. With such a masking, a more severe consequence can be induced such as cascading failures. On the other hand, the physical attack cannot be stealthy to the operator [64]. In [64], the two types of CCPAs in electric power systems were studied, including the coordination between load redistribution (LR) attack and attacking generators, and the coordination between LR attack and attacking lines. Bilevel optimization problems were generated to formulate these CCPAs, where the defense at the lower level attempts to increase load reduction while the attacker at the upper level strives to minimize it. Further discussion on the misleading function of a cyberattack in CCPAs was presented in [65], where the attacker alters the measurement data to create a fake overload event that leads the dispatcher to believe that a transmission line that is routinely running is overloaded. Consequently, the irrational dispatching strategies employed by the dispatcher would negatively impact the profitability of the CPS, thereby compromising its operational efficiency. A defense strategy for CCPAs was employed in [66] by utilizing distributed flexible AC transmission system devices and actively perturbing the grid’s transmission line reactances, such that the necessary knowledge that the attackers aim to mask the effects of the physical attack can be made invalid. A tri-level optimization model was formulated in [67] to describe the interactions among defender, attacker, and operator in a 24-h horizon. The proposed model is capable of analyzing the protective measures against attacks that result in component failure within a matter of hours, as opposed to previous studies that focused on attacks that resulted in component degradation over several days.

Additionally, in the realm of cyberattacks, the attacker may consider not only the coordination between cyber and physical attacks, but also the interaction between availability and integrity attacks in order to maximize the impact of the attack. CCPAs combined with DoS attacks in smart grids were studied in [68], where mathematical models were constructed for two types of the CCPAs. With regards to the stealthy one, it has been demonstrated that the attack objectives can be accomplished with fewer resources if DoS signals are also introduced. In the case of the non-stealthy type, it has been established that significant effects can be achieved, and a bilevel optimization technique was employed to determine an optimal attack strategy.

By a brief survey of CCPAs, one could know that such attacks differ dramatically from the previous works that focused solely on hybrid attacks in cyberspace. It is crucial to recognize that a new type of attack has become increasingly prevalent in recent years, especially in domains such as smart grids. CCPAs are characterized by integrating both cyber and physical attacks, making them distinct from traditional attacks. Thus, traditional cybersecurity measures may not be sufficient to protect against CCPAs since they involve physical attacks. Hence, there is a need to integrate physical security measures with cybersecurity measures to ensure comprehensive protection. This will call for multidisciplinary approaches to work together to develop effective countermeasures to CCPAs. A deeper understanding is also required to reveal the technical, organizational, and human factors contributing to CCPAs, which can contribute to effective prevention and mitigation strategies.

## 3. Recent Proactive Defense Strategies for Securing Complex Cyber–Physical Networks

In the above section, the modeling of several cyberattacks and corresponding reactive defense strategies have been briefly surveyed. However, when confronted with more sophisticated and smart attacks whose characteristics or attack parameters cannot be predicted in advance, the traditional reactive defense strategies may only have limited functions. Thus, it is urgently desirable to develop secure control algorithms of a proactive nature that can sustain or recover a specific system’s performance in the face of unforeseen attacks. To mitigate cyberattacks’ impacts on cooperative systems, a range of resilient cooperative control algorithms have been presented in the literature. These methods mainly need specific knowledge about the considered attack, such as the upper bound of the maximum number of attacks, the attack’s duration, or the attacker’s budget. Furthermore, some restrictions on the communication network architecture are needed for certain types of cyberattacks. For instance, when a certain number of agents are attacking and being adversarial, with a specific resilient control scheme, such as mean-subsequence reduced (MSR) algorithms, sufficient conditions regarding the robustness of graphs are derived aiming to mitigate the negative impacts from the adversarial and resilient consensus [13]. Notably, MSR and double-integrator position based MSR (DP-MSR) algorithms were investigated in [69], where some necessary and sufficient conditions regarding the topology restrictions were given for resilient consensus of NASs with time-varying communication graphs and under scenarios with misbehaving agents. A notion of joint robustness was innovatively proposed to characterize the graph robustness properties.

This section will first report some recent works on the active defense from the network or topology view, by elaborately designed dynamic networks or communication networks, or activating effective topology reconstruction strategies to mitigate the negative impacts induced by the attacker. Additionally, with ideas brought from the hybrid system and computer science, research has investigated the application of the concepts of active switching-based control and moving target defense, which can proactively adopt alternate control techniques to reduce stealthiness, increase the cost of attacks, and limit their impacts.

### 3.1. Secure Defense Leveraging Network or Topology Design

Firstly, a resilient control algorithm was proposed in [70] for leaderless consensus by artificially constructing a virtual network. The main feature of such a virtual-network-assisted framework is that no assumptions on the maximum number of attacks is required. Different kinds of attack cases were also handled based on this strategy, such as attacking actuator, sensor, and/or communication network [71,72]. Furthermore, many researchers employed this method in different applications with various network settings, including formation containment control [72], networked heterogeneous systems [71], resilient control of AC microgrids [73], distributed economic dispatch [74], and connected vehicles [75]. The event-trigger control version was further given in [76] for general linear multi-agent systems to realize the leader–follower consensus under unknown attacks. For a dynamic time-varying leader, the introducing of a virtual network could ensure that followers track the leader’s state with tolerable errors [77]. In [78], the authors explored the attack scenario where an adversary can also temper the information exchanged among the virtual network; a new virtual network with time-varying weights was given to make it more difficult for the attacker to launch a stealthy attack compared to the virtual network with constant weights:

(8) $$\begin{array}{cc} \dot{x}\left(t\right) & =-L_{s}\left(x-d\right)+\beta L_{s}\Gamma \left(t\right)z,\\ \tilde{z}\left(t\right) & =-Hz-\beta \Gamma \left(t\right)L_{s}x, \end{array}$$

with matrices given by

(9) $$\begin{array}{cc} H & =L_{s}+I_{n},\\ \Gamma (t) & =\mathrm{diag}\{{\left[\alpha_{1}\left(t\right),\cdots ,\alpha_{n}\left(t\right)\right]}^{T}\}, \end{array}$$

where $z\left(t\right)\in R^{n}$ is the state of the introduced virtual network, β is the positive constant parameter to be determined (usually taking sufficiently large values), $L_{s}$ the Laplacian matrix corresponding to the original cooperative system, $d\left(t\right)\in R^{n}$ denotes the unknown exogenous injections, and $d_{i}\left(t\right)\neq 0$ represents that the information received from node *i* is being compromised and the local state feedback of node *i* is corrupted. The dynamic weights of the Laplacian matrices associated with the virtual network $\Sigma_{h}$ are given by Γ(t) with $\alpha_{i}>0$.

Some work seeks to design a more resilient network structure to resist potential general attacks by introducing some indexes and formulating the corresponding optimization problems. A current work [79] gave a structural survivability index regarding the variance of node degree, based on which a two-stage optimization approach was derived for the communication topology design of microgrids, where resilience and network dynamic performance are jointly optimized.

By utilizing the adaptive method, an adaptive consensus protocol was proposed in [80] to coordinate the states of incremental costs in the distributed economic dispatch when unknown uncertainties exist in the communication network:

(10) $$\begin{array}{cc} {\dot{\zeta }}_{i}\left(t\right) & =2c\sum_{j\in N_{i}}\left[a_{ij}\left(t\right)+\omega_{ij}\left(t\right)\right]\left[\zeta_{j}\left(t\right)-\zeta_{i}\left(t\right)\right],\\ {\dot{a}}_{ij}\left(t\right) & =h_{ij}{\left[\zeta_{i}\left(t\right)-\zeta_{j}\left(t\right)\right]}^{2},\;\;j\in N_{i}, \end{array}$$

where $\omega_{ij}$ are the unknown uncertainties, $h_{ij}=h_{ij}$ are the positive constants related with the adaptive speed of time-varying weighted adjacency matrix $A\left(t\right)={\left[a_{ij}\left(t\right)\right]}_{N\times N}$. It is assumed that $\omega_{ij}\left(t\right)=\omega_{ji}\left(t\right)$, $j\in N_{i}\left(t\right)$, i,j=1,2,⋯,N, and

(11) $$|\omega_{ij}\left(t\right)|\leq \sigma_{ij},\;\;\forall t\geq 0,$$

with $\sigma_{ij}$ being positive scalars which could be unknown. Thus, the introduced uncertainty could also be used to describe edge manipulation attacks with unknown attack intensity. It was rigorously proved that optimal economic dispatch can be solved by such an adaptive weight-adjustment technique. Further considering the event-triggered framework, the countermeasures for such an edge attack case were investigated in [81]. It was assumed that the unknown uncertainties induced by the malicious cyberattacks on communication edges alter the ideal weights $a_{ij}$ into ${\tilde{a}}_{ij}\left(t\right)$ in both distributed control protocols and also the event-triggered conditions. First, the consensus-based event-triggered protocol was given as:

(12) $$\begin{array}{cc} {\dot{x}}_{i}\left(t\right) & =2\gamma_{i}\sum_{j\in N_{i}}{\tilde{a}}_{ij}\left(t\right)\left(x_{j}\left(t_{k_{j}\left(t\right)}^{j}\right)-x_{i}\left(t_{k_{i}\left(t\right)}^{i}\right)\right),\\ {\tilde{a}}_{ij}\left(t\right) & =c_{ij}\left(t\right)+\omega_{ij}\left(t\right),\\ {\dot{c}}_{ij}\left(t\right) & =h_{ij}{\left({\tilde{x}}_{i}\left(t\right)-{\tilde{x}}_{j}\left(t\right)\right)}^{T}\left({\tilde{x}}_{i}\left(t\right)-{\tilde{x}}_{j}\left(t\right)\right), \end{array}$$

with $x_{i}\left(t\right)\in R^{n}$, and there exists a nonnegative constant $\bar{\omega }$ such that $\omega_{ij}\left(t\right)\leq \bar{\omega }$, with event-trigger condition implemented by agent *i* given by:

(13) $$t_{k_{i}\left(t\right)+1}^{i}=\inf\left\{t|t>t_{k_{i}\left(t\right)}^{i},\psi_{i}\left(t\right)\geq 0\right\},$$

with event-trigger condition

(14) $$\psi_{i}\left(t\right)=\left[4\rho_{2}+\eta_{1}\left(12+4\rho_{1}\right)\right]\sum_{j\in N_{i}}{\tilde{a}}_{ij}e_{i}^{T}e_{i}\left(t\right)-\sigma_{2}\sum_{j\in N_{i}}{\tilde{a}}_{ij}{\left[{\tilde{x}}_{i}\left(t\right)-{\tilde{x}}_{j}\left(t\right)\right]}^{T}\left[{\tilde{x}}_{i}\left(t\right)-{\tilde{x}}_{j}\left(t\right)\right]-\mu e^{-\nu t},$$

with $e_{i}\left(t\right)={\tilde{x}}_{i}\left(t\right)-x_{i}\left(t\right)$ being the estimation error, and other free-weight parameters were also determined such that the stability of the closed-loop system can be achieved and Zeno behavior could be avoided.

### 3.2. Secure Defense by Topology Reconstruction

Research has been conducted on network topology reconstruction after cyberattacks, such as the ability to recover from an attack quickly being crucial in maintaining the stability and security of a network. These attacks often result in unpredictable and complex network structure changes, making them difficult to defend against. Network topology reconstruction aims to quickly and accurately detect these changes and restore the network to its normal state. Various methods have been utilized to achieve this goal, including machine learning algorithms and network analysis techniques.

In [82], for an attack that targets the cutting nodes and edges among the directed nonlinear multi-agent systems, the corresponding recovery strategies to reconnect these components and fast convergence speed were developed. An objective function was selected to identify the unique solution:

(15) $$f_{G}\left(\tilde{G}\right)=\frac{a(\tilde{G})}{1+\sum_{v_{i}\in V}\left(d_{i}\left(\tilde{G}-d_{i}\left(G\right)\right)\right)},$$

where $a(\tilde{G})$ is the algebraic connectivity of the recovered graph $\tilde{G}$. The attack that may cause the cut-agent and cut-link failures in multi-agent systems was studied in [83]. Different measures were taken to restore the connectivity of multi-agent systems in the event of different failures with the aim of restoring consensus among agents. The proposed recovery strategies focus on improving the convergence speed of the network in reaching consensus as the main goal and revealed the trade-off between the convergence speed and robustness of the resulting networks. In [84], the authors present a consensus recovery algorithm for multi-agent systems with nonlinear dynamics that can handle node failure. The algorithm utilizes local information of the failed node and its neighbors to re-establish connections and recover consensus. The algorithm has been proven to be effective through rigorous mathematical proof and has the advantage of preserving the consensus magnitude before failure even after recovery. A recovery approach was proposed in [85] to protect MASs from the impact of agent failure in hostile environments where repair is not possible. The approach aims to achieve faster consensus by creating new connections with agents with high node degrees. A cost function is introduced to maximize the uniqueness of the recovery solution. The authors provide mathematical proof for the convergence of the algorithm, showing that, after failure, the algorithm leads to faster consensus by improving the network’s algebraic connectivity.

In the application of formation control, the topology recovery problem for multi mobile robots was investigated in [86]. A connectivity recovery scheme was proposed under the situations where the communication link has failed. Two theorems were obtained to illustrate the guarantees for the success of topology recovery. The problem of robust synchronization control in complex cyber–physical networks that face mixed attacks was explored in [87]. The control input signals and information channels were considered to be independent and both can be compromised by frequent attacks. To address this, a repairing cell is immediately activated to restore the communication topology and sufficient conditions were established to ensure robust synchronization. In [3], the authors studied an efficient secure control technique based on “reconstruction + repairing” in the face of malicious node attacks to achieve global pinning synchronization of complex CPS networks. Two attack mitigation strategies for distributed event-triggered control for TCL regarding the two edge manipulation attack cases were proposed, respectively, in [88]. According to the degree of attack intensities, two categories of edge manipulation attacks were first discussed. For the mild edge attack case where the communication network’s connectivity is preserved and some communication links may have been tampered with, a mitigation strategy utilizing the superposition graph concept was given to improve the convergence rate of realizing fair power state sharing. For the severe attack case where the connectivity of the communication network fails to be retained, two alternatives from the building-cluster level and within-building level were newly designed to reconstruct connected communication topologies. For the distributed consensus tracking problem of networked agent systems with directed topologies under DoS attacks, two connectivity restoration strategies to reduce the impacts of the attack in the cyber layer were developed [24]. According to the intensity of the DoS attack, the strategy with distributed perspective or that with a global perspective can be freely chosen. The strategy with distributed perspective includes the method of active reserved links and adding bidirectional links connecting all of these isolated nodes to their neighbors, while the one with a global perspective can be applied by adding bidirectional links between each pair of nodes to resist severe attacks.

In [89], the authors investigated a distributed framework for network recovery for MASs. Based on the algebraic connectivity (reflecting the reconstructed network’s performance) and $|{\tilde{E}}_{\phi }\left(t_{k}\right)|$ (the set of added edges reflecting the reconstruction budget), the evaluation function was proposed as:

(16) $$P\left(S\right)=\frac{\lambda_{2}\left(G_{k}\right)-\lambda_{2}\left({\tilde{G}}_{k}\right)}{1+|{\tilde{E}}_{\phi }\left(t_{k}\right)|}$$

where *s* is the type of reconstruction strategy. The rest of the agents without attack will reconstruct the network and restore the connectivity by following the proposed communication strategy in a distributed fashion. A method for identifying faults in uncertain MASs was proposed in [90]. The method includes a filter-based approach and a topology reconstruction-based fault isolation strategy to improve the fault identification performance. The use of a cooperative fault detection system was proposed to enhance the effectiveness of the proposed topology reconstruction and logic-table-based fault identification scheme. A learning strategy was employed in [91] to restore connectivity coping with loss of inter-agent communication in the formation control multi-agent system. This was a decentralized learning technique for mobile agents where each agent uses only local information and trains to handle the risk of connectivity loss. This training involves agents making coordinated movements based on consensus dynamics, with a lost agent able to predict its way back to the fleet while other agents continue their formation.

The reconstruction of network topology after an unknown cyberattack is crucial to cybersecurity for complex cyber–physical networks. The above brief survey in this subsection aims to ensure that the network returns to its normal state in the shortest possible time. Attacks on network structures can lead to the emergence of unpredictable changes that require timely detection and remediation. The use of machine learning algorithms and network analysis techniques demonstrates their particularly promising roles in facilitating fast and accurate network topology reconstruction. Future research could focus on enhancing these methods’ efficiency, effectiveness, and distributed framework for large-scale complex cyber–physical networks, and exploring new avenues to achieve faster recovery times.

### 3.3. Secure Defense with Active Switching

A subset of stealthy attacks known as zero-dynamics attack (ZDA) has gained much importance in recent years. The attacker’s objective consists of two components: one is avoiding the detection by maintaining the monitoring outputs unchanged, so it is “stealthy” and, more significantly, it aims to alter the system to accept the tampered bad data that differs dramatically from the real data such that some specified system’s states can deviate from expected values. The two most notable characteristics of earlier works are either placing restrictions on the size of the misbehaving-agent set and the connectivity of network topology or demanding the defender side be aware of misbehaving agents and the attack starting time in order to detect attacks. A topology switching strategy technique was suggested in [92] to eliminate the above restrictions and unreasonable assumptions. A defense strategy that consists of strategic topology switching and strategic output observing was proposed to combat this difficult stealthy attack in coupled harmonic oscillators. With this defense strategy, cooperative zero-dynamics attacks are still possible but do not affect steady-state value or system stability. For general linear systems, the topology switching technique was developed in [93] to solve the issue of which topologies to adopt to identify ZDA. Based on Luenberger observers, an algorithm to determine the switching signal of candidate communication topology was proposed. The essence of the defense method suggested above is to alter system dynamics by switching communication topology, so that the ZDA is no longer achievable. Generally, the corresponding dynamics of system under attacks are given by:

(17) $$\begin{array}{cc} \dot{\tilde{z}}\left(t\right) & =A\tilde{z}+Bg\left(t\right),\\ \tilde{y}\left(t\right) & =C\tilde{z}+Dg\left(t\right), \end{array}$$

where g(t) is that attack signal.

The attack signal with attack-starting time instant ρ:

(18) $$g\left(t\right)=\left\{\begin{array}{c} ge^{\eta (t-\rho )},t\in \left[\rho ,\infty \right),\\ 0,\;\;\;\;\;\;\;\;\;\;\;\;\;\;\;\;\;\;t\in [0,\rho ), \end{array}\right.$$

is called the zero-dynamics attack, if $0_{\bar{n}}\neq \tilde{z}\left(0\right)-z\left(0\right)\in R^{\bar{n}}$, $0_{\bar{o}}\neq g\left(\rho \right)\in R^{\bar{o}}$, ρ≥0 and η∈C satisfy:

(19) $$\begin{array}{cc} \tilde{z}\left(0\right)-z\left(0\right) & \in \ker(O),\\ \left(\begin{array}{c} e^{A\rho }\left(\tilde{z}\left(0\right)-z\left(0\right)\right)\\ -g(\rho ) \end{array}\right) & \in \ker\left(\begin{array}{cc} \eta 1_{\bar{n}\times \bar{n}}-A & B\\ -C & D \end{array}\right), \end{array}$$

where $O≜{\left[C^{T},{\left(CA\right)}^{T},\cdots ,{\left(CA^{\bar{n}-1}\right)}^{T}\right]}^{T}$. It also verified that, under the ZDA (18) and (19), the states and monitored outputs of original system satisfy:

(20) $$\begin{array}{cc} y(t) & =\tilde{y}\left(t\right),\mathrm{for}\;\mathrm{all}\;\;t\geq 0,\\ z(t) & =\left\{\begin{array}{c} e^{At}\tilde{z}\left(0\right),\;\;\;\;\;\;\;\;\;\;\;\;\;\;\;\;\;\;\;\;\;\;\;\;\;\;\;\;\;\;\;\;\;\;\;\;\;\;\;\;\;\;\;\;\;\;\;\;\;\;\;t\in \left[0,\rho \right),\\ z\left(t\right)+\left(\tilde{z}\left(\rho \right)-z\left(\rho \right)\right)e^{\eta (t-\rho )},t\in \left[\rho ,\infty \right), \end{array}\right. \end{array}$$

In [94], the authors investigated the switched-based secure control design for attacks with intermittent communication faults satisfying specified frequency and duration restrictions. By assuming that the switching law for faults is unknown to the considered CPS, a switching law for updating the control parameters was developed based on the designed inequality, including the Lyapunov-form functions. Then, sufficient conditions were given, under which the original system with the switched-based control laws could realize global asymptotic stability. A switching-like event-triggered communication strategy is well-built to increase communication efficiency by fully utilizing the DoS attack capabilities in [95]. With the acknowledgment character (ACK) technique, the plant can know whether the transmitted data are lost. Then, one could actively select the proper event-triggered parameters to minimize the amount of communicated packets. The tracking control problem for multi-agent systems under sensor attacks with *s*-sparse vector was studied in [96]. A cooperative attack tolerant tracking control strategy based on a switching scheme was designed to ensure that the global tracking error maintained uniformity and was ultimately bounded. To search all of the attacked sensors, a common switching function matrix is employed for each node, such that the matrix switches in response to the switching index change when the attack is detected.

Note that the traditional control theoretic framework usually produces a stabilization protocol that is passive in the face of cyber threats and conservative in terms of the specified degree of resilience. A fully heterogeneous interconnected MAS is described in [97], where the modeling uncertainties and nonvanishing perturbations on the agent layer exist, and distributed DoS attacks happen over the control layer. No assumptions were made to limit the DoS attack class by average dwell time or probability distribution. A distributed switching control protocol with candidate communication graphs was proposed as follows:

(21) $$\begin{array}{cc} u_{i}\left(t\right) & =\sum_{j\in N_{i}^{c\sigma (t)}}a_{ij}^{c\sigma (t)}\left(v_{i}\left(t\right)-v_{j}\left(t\right)\right)+s_{i}^{c\sigma (t)}v_{i}\left(t\right),\\ v_{i}\left(t\right) & =K_{i}x_{i}\left(t\right), \end{array}$$

where i=1,2,⋯,M, M≤1 denotes the number of control sublayers, $a_{ij}^{c\sigma (t)}\geq 0$ denotes the edge weights of a control sublayer’s undirected graph with regard to the switching signal σ(t), $s_{i}^{c\sigma (t)}>0$ denotes that there exists selfloop weights of control node *i*, and $s_{i}^{c\sigma (t)}=0$ otherwise. $K_{i}$ is the feedback control gain to be designed for agent *i*. When the control layer employed the arbitrarily fast switching, it has been proved that the robust input-to-state stability (ISS) of the two-layer interconnected MAS with the proposed proactive cyber defense strategy can be assured.

Wind energy systems in power grids including the SCADA system, communication networks, wind farm local servers, etc., can be viewed as a typical networked cyber–physical system, and a cyber-aware robust and resilient switching controller was given in [98]. The following output feedback controller based on a Markov jump linear system was proposed:

(22) $$\begin{array}{cc} {\dot{x}}_{F} & =A_{F}\left(\vartheta_{t}\right)x_{F}\left(t\right)+B_{F}\left(\vartheta_{t}\right)y\left(t\right),\\ u(t) & =C_{F}\left(\vartheta_{t}\right)x_{F}\left(t\right), \end{array}$$

where $A_{F}\left(i\right)=A_{Fi}$, $B_{F}\left(i\right)=B_{Fi}$, and $C_{F}\left(i\right)=C_{Fi}$ are system matrices to be determined by solving the derived linear matrix inequalities (LMIs), $x_{F}\left(t\right)$ is the designed controller’s state, i∈F={1,2,⋯,S}, and $\left\{\vartheta_{t},t\geq 0\right\}$ is a right-continuous Markov chain regarding the various operating conditions of wind energy system. Then, a system-of-systems approach for a holistic design was developed, where the system robustness, security, and resilience were all taken into account. A strategic defensive mechanism for the cyber-layer operator was developed by introducing the non-cooperative game theory. It was proved that the proposed switching controller is resilient and the system-of-systems equilibrium was calculated utilizing an iterative technique by bi-linear programming.

### 3.4. Moving Target Defense for Securing Complex Cyber–Physical Networks

Moving Target Defense (MTD) has surfaced as a fundamental approach for increasing uncertainty regarding a system’s state or execution, prohibiting attackers from having predictable effects with their attacks. The core idea of MTD originated from the computer science field by cyber resilience or agility techniques, which proactively change network communication paths to make the routing topology difficult to predict and, thus, rescue the attacked traffic [99]. By introducing uncertainty into the parameters of the complex cyber–physical networks, MTD has been applied in several CPS scenarios in recent years and most of these applications can be roughly clarified into three types: (1) attack detection; (2) complicating and raising the cost of attackers gathering system information and executing successful attacks; and (3) mitigating the impact of cyberattacks.

Firstly, in the application field of detecting attacks, the security of an MTD strategy for improving the detectability of cyberattacks was analyzed in [100]. The MTD algorithm is effective in detecting a particular class of stealthy attacks. These attacks are characterized by the attacker’s knowledge of the system dynamics, detection strategies, and access to all sensors and control inputs. A sufficient condition has been established that allows for detecting these stealthy attacks through implementing the proposed MTD algorithm while simultaneously minimizing the impact of such attacks. A detection method based on the MTD framework was proposed in [101], in which data regarding the system’s changing operating point was utilized to cluster measurements based on their transfer function characteristics. A series of similarity tests between measurements within the same cluster are used to find the attacks’ occurrence. The suggested technique may identify intelligent cyberattacks that are knowledgeable in system parameters, models, and detection rules. In [102], a switching-based MTD strategy was developed to detect malicious FDI attacks described by the average dwell-time feature, an ET mechanism was also applied to reduce the cost of data transmission. Even if attacks may remain stealthy when the MTD mechanism is adopted, the impacts of these stealthy attacks on the system performance can be kept to a minimum level. To defend against deception attacks, a MTD strategy based on converters with perturbed primary control gains was proposed in [103]. The impact of perturbed control gains on the voltage stability in DC microgrids was studied, and sufficient conditions of the perturbation magnitude and frequency were derived to ensure voltage stability. It was shown that detectability could be improved under such a scheme and conditions to successfully detect such attacks were provided. By employing the distributed flexible AC transmission system (D-FACTS), the correlation between the developed MTD and detection for FDI attacks was demonstrated in [104]. The results also offered a heuristic technique to determine a near-optimal solution for the deployment of D-FACTS devices to decrease the amount of measurements the attacker can change after MTD.

By causing additional difficulty or cost for attackers, MTD has also been applied in both the estimation and control of complex CPSs. In applying the power grid, the state estimations are often utilized to generate optimal power flow and perform contingency analysis; false estimations will directly impact the economic and system operations. In [105], randomization was mainly on the set of measurements in state estimation and line admittances were applied such that information uncertainty would be generated, which contributes to the limitation of the potential attack space of undetected false data injection attacks.

MTD was also used in [106] for AC state estimation, where an extended MTD approach was presented by changing series reactance and parallel susceptance of lines in coordination. Furthermore, how the proposed MTD method influences the electricity market was also discussed. The cost of system defense was modeled as integrating the locational marginal price, the variation of active power loss, and the cost of devices for executing MTD. The optimal MTD parameter was given balancing the defense unity and cost.

For a secure control perspective, a proactive and reactive defense mechanism by applying the idea of MTD was proposed in [107], where a stochastic switching structure is designed to dynamically and continually adjust system settings while impeding the attacker’s capacity to perform system reconnaissance successfully. By introducing the candidate actuating operating mode $B_{i}$, $i\in \{1,\cdots ,\mathrm{card}\left(B_{c}\right)\}$, the following linear system with candidate controllers can be derived:

(23) $$\dot{x}\left(t\right)=Ax\left(t\right)+B_{i}u_{i}\left(t\right),\;t\geq 0,$$

with $B_{c}=\left\{B_{j}\in 2^{B}:\mathrm{rank}\left(\left[B_{j}\;AB_{j}\;\cdots \;A^{n-1}B_{j}\right]\right)=n\right\}$. Based on maximizing the information entropy, which is commonly used as a standard practice in MTD, the probability $p_{i}$ that each controller is employed is developed as:

(24) $$p_{i}=e^{\left(-\frac{V_{i}^{*}}{\epsilon }-1-\epsilon \log\left(e^{-1}\sum_{i=1}^{N}e^{\frac{V_{i}^{*}}{\epsilon }}\right)\right)},$$

where

(25) $$V_{i}^{*}\left(x\left(t_{0}\right)\right)=\min_{u_{i}}\int_{t_{0}}^{\infty }\left(x^{T}Q_{i}x+u_{i}^{T}R_{i}u_{i}\right)d\tau ,\;\forall x\left(t_{0}\right),$$

with $Q_{i}\geq 0$, R>0, and ϵ≥0 reflecting the weight on unpredictability during the optimization process. It is proved that under attack-free scenarios, an asymptotically stable equilibrium point for every switching signal can be ensured if the average dwell time is bounded by a given number. The detection signal with a certain time window *T* was also designed:

(26) $$e\left(t\right)=\hat{V_{i}}\left(x_{c}\left(t-T\right)\right)-\hat{V_{i}}\left(x_{c}\left(t\right)\right)-\int_{t-T}^{t}\left(x_{c}^{T}Q_{i}x_{c}+u_{i}^{*T}R_{i}u_{i}^{*}\right)d\tau .$$

where $\hat{V_{i}}\left(\cdot \right)=x_{c}^{T}P_{i}x_{c}$, with $x_{c}$ denoting the state at the sampling instants, and $u_{i}^{*}\left(x\right)=-R_{i}^{-1}B_{i}^{T}P_{i}x$. The core idea therein is that the switching probabilities are chosen to optimize the entropy induced by the switching strategy while guaranteeing that the controller cost is kept to a minimal level. The event-triggered control version was further developed in [108]. The event function was given to determine the triggered instants and the inter-event time’s lower bound was also derived concerning the event-triggered parameters. A corresponding intrusion detection algorithm was then given to reveal actuator intrusions. Immediately after the results in [108], the metaheuristic method based on the particle swarm optimization was further used in [109] to tune the controllers therein and improve their control performance.

Randomized switching methodology under the adaptive framework was developed in [110] where, at run-time, the performance of several parallel controller implementations were examined to adjust the probability of switching to each controller. Different from simple switch strategies, such as round-robin protocol, a time-division multiplexer was introduced, where the misbehaving controller nodes can be turned off based on observation of their performance but the controller will be rechecked for evaluation after some point, even if the likelihood of switching to a certain controller becomes low. Ref. [111] proposed the moving target defense strategy based on a completely model-free framework for a discrete-time system. Approximate dynamic programming (ADP) was used to develop intrusion detection based on Bellman error, such that policy for optimal regulation and tracking can be learned. Switching rules are leveraged to force proactive and reactive defense mechanisms defending against actuator and sensor attacks and guarantee the equilibrium point’s stability. A MTD and reinforcement learning strategy was proposed in [112], where several controllable pairs that can alter the system’s dynamics were introduced with unpredictable switching probabilities. Attack detection and isolation methods were developed to accurately locate and exclude the compromised actuators from the candidate sequence and, when no subsystem can be utilized, a defense control based on reinforcement learning was also given. In [113], the authors investigated the security control for time-varying delay systems. The Lyapunov stability analysis approach is used to jointly generate sufficient requirements for the stability of the observer and control system, in which a moving defensive strategy is based on anomaly detection and random switching, along with an optimization problem for computing the correct switching probability of each potential actuator–controller combination.

Different from work [107] focusing on the entropy, considering the minimizing of impacts caused by the attack, random communication topologies for multi-vehicle systems as a MTD were used in [114], while also maintaining control performance under attack-free cases. The attacker under consideration can intercept interactions between two agents and falsify the information being conveyed. An MTD is designed by introducing the time-varying graph G(k)=(V,E(k),A(k)) with $a_{ij}\left(k\right)=1$ being a Bernoulli random variable with probability $0\leq p_{ij}\leq 1$ and the connection probability matrix $P\in R^{n\times n}$ can be defined with:

(27) $$\begin{array}{cc} P_{ij} & =\left\{\begin{array}{c} p_{ij},\;i\neq j,\\ 0,\;i=j. \end{array}\right. \end{array}$$

For the modified Erdös–Rényi graph with 0<p<1, a necessary and sufficient condition for the union of graphs was derived such that the introduced MTD strategy does not influence the consensus state under an attack-free situation, and the expected convergence rate is explicitly given by

(28) $${\bar{R}}_{MTD}=1-\sqrt{1-p\frac{\mu_{2}}{\mu_{n}}},$$

with $\mu_{1}=0\leq \mu_{2}\leq \cdots \leq \mu_{n}$ being the eigenvalues of supergraph $G_{T}$ (with all possible communication links among agents). More interestingly, by introducing the attack impact index as $\lim_{k\to \infty }∥x\left(k\right)-x_{c}∥$, the impact of the attack can be given by

(29) $$\bar{F}=\frac{p}{\mu_{n}}\sqrt{n(2p^{2}+2p+1)}.$$

It is revealed that trade-offs exist between the convergence rate and the attack impacts. Interestingly, the results can guide system designers so that the amount of possible communication channels can be increased such that smaller *p* can be picked without causing substantial performance reduction while ensuring strong resistance to attacks. A MTD strategy that leverages the versatility of IoT networks to reinforce the security of CPSs was presented in [115]. This strategy entails the replication of crucial sensory and control signals, leading to the creation of two layers of unpredictability, thus diminishing the capability of adversaries to conduct successful cyberattacks without affecting the normal performance of the system.

From the above brief survey in this subsection, one can see that this proactive technique involves introducing uncertainty into the parameters of complex networks, thereby rendering them more resilient to attacks by three main reasons: Firstly, MTD can be utilized for the purpose of detecting attacks. Secondly, it can be employed to increase the difficulty and cost for attackers attempting to obtain system information and execute successful attacks. Finally, MTD is used to mitigate the impact of cyberattacks. Further research is required to explore the effectiveness and limitations of MTD in the context of different scenarios. It is crucial to identify the potential risks and challenges associated with this approach, such as the possibility of introducing unintended vulnerabilities or impacts into the system. Additionally, future studies may investigate the optimal implementation of MTD techniques, considering the system’s specific characteristics under certain operating situations. There is also a need to develop standardized methodologies for evaluating the effectiveness of MTD approaches, which can help compare the performance of different MTD techniques and assess their suitability for different applications.

## 4. Conclusions and Discussions

This survey has examined the most recent advancements and proactive defense strategies in complex cyber–physical networks. For single-type classical cyberattacks, advances for modeling and tackling DoS attacks and FDI attacks have been mainly explored. The newly emerging hybrid cyberattacks that are more stealthy and collusive have been investigated in depth, including those that only occur in cyberspace and the coordinated cyber–physical attacks that combine the benefits of physical and cyberattacks. Based on topology and control viewpoints, several existing defense strategies have been reviewed for a more proactive defense goal. The topological design enables the defender to anticipate future attacks, while the reconstruction process can contribute to the recovery from inevitable attacks. Works that employ the ideas of active switching-based control and moving target defense have also been addressed. The defense can adopt alternate control strategies proactively to minimize stealthiness, raise the attack budget, and limit attack impact.

Despite the abundant reporting of significant outcomes in recent years, it is imperative to acknowledge the critical limitations of current secure control methods due to their stringent assumptions and particular demands. The widespread adoption of artificial intelligence (AI) technology, coupled with the continuous evolution of network defense, has opened up new avenues for future research that offer substantial theoretical and practical significance. The following areas are particularly intriguing and warrant further investigation.

(1) AI-aided secure control and applications. Many existing secure control schemes assume that either the system knowledge is known to the attackers or that the attack scheme is known to the defenders. However, the presence of system uncertainties, either internal or external, makes these assumptions unrealistic in many real complex cyber–physical networks. AI techniques, such as machine learning, and reinforcement learning, when combined with existing system dynamics models, could offer a promising way to address such environmental uncertainties and improve the security of CPSs.
(2) Game-theoretic framework for secure control. The utilization of game theory in the secure control design of CPSs involves modeling an attacker and a defender as rational decision-makers, thus enabling the analysis of their interactions to determine optimal strategies for both sides. The defender strives to create a secure control system that reduces the likelihood of a successful attack, while the attacker endeavors to compromise the system’s normal functioning. One challenge is the intricate nature of complex cyber–physical networks. The interactions between the physical and cyber components will complicate the prediction of the effects of certain control actions, and also poses difficulties in designing a control system when both security and performance objectives are considered. Game theory provides a valuable framework for the security of CPSs and technical challenges still remain in addressing the uncertainty and complexity intrinsic therein.
(3) Distributed mitigation and recovery approaches for complex cyber–physical networks. It is imperative to note that, in order to implement effective recovery or reconstruction strategies in complex cyber–physical networks, a significant amount of global information about the system is needed in most existing works. However, such requirements violate the distributed nature of complex cyber–physical networks and limit the potential applications of mitigation and recovery methods. Hence, it is essential to conduct in-depth discussions on how to realize the mitigation and recovery approaches in a fully distributed manner, optimize the recovered network while considering practical cost indexes, and consider the possibility of various sophisticated cyberattacks with attacking impacts.

## Figures and Tables

**Table 1:** Brief summary and comparisons of several existing survey papers.

Year	References	Main Focus or Contributions	Scenarios Considered
2021	[11]	Deception attack, DoS attack, Physical fault estimation, detection and diagnosis, and fault tolerant control	Homogeneous/heterogeneous multi-agent systems
2022	[16]	Availability, integrity, and confidentiality attacks for time-driven and event-driven systems	Cyber physical system
2022	[12]	DoS attack, deception attack, attacks on agents	Multi-agent systems
2022	[13]	Mean subsequence reduced (MSR) algorithms for FDI attack, DoS attack	Multi-agent systems
2022	[14]	DoS attack, FDI attack, replay attack, attack space	Networked control systems
2022	[17]	DoS attack, deception attack, hybrid attack, event-triggered secure control	Networked control systems
2022	[15]	Deception attack	Networked control systems
	This survey	Single type classical cyberattacks, hybrid intricate cyberattacks, recent proactive defense strategies from topology and control view	Complex cyber–physical networks

## Data Availability

Not applicable.
